# The Book World of Medicine and Science

**Published:** 1906-06-23

**Authors:** 


### The Book World of Medicine and Science.

Notes on General Practice. By S. M. Hebblethwaite, M.D.Lond. (Scientific Press.)

This is an interesting book. One often feels that there is need for men possessing wide experience, gained in the general practice of their profession, to say more than they do about facts of medicine and surgery as they present themselves to the busy man, who is at the same time the man of thought and intelligence. Dr. Hebblethwaite quotes Ruskin as saying that art is the outcome of man's pleasure in labour. It is the idea that much of the interest of medical work is lost when connection with a hospital is severed that makes so many desire to obtain staff appointments. But while hospitals afford greater opportunity for adding to knowledge than general practice, there are aspects of both life and medical work which never come under the notice of those who do not have their daily round of visits to varied homes. A comment may be made upon one physical sign referred to in cases of abdominal injury or disease. That is absence of liver dullness as an indication that there is gas in the peritoneal cavity. Dr. Hebblethwaite considers—and we believe rightly—that the absence of liver dullness is not necessarily an indication of rupture of the intestine or of perforation of a gas-containing viscus. The absence of liver dullness is most commonly due to distension of the intestines, which press upwards the liver. It is the resonance of gas inside the distended intestines, not the resonance of gas outside them, which causes the liver dullness to disappear. In other instances, of which possibly a case referred to by Dr. Hebblethwaite was an example, the transverse colon is situated over the upper surface of the liver. It may become displaced, there, as suggested by Sir Wm. Bennett, by the constriction of an abdominal belt.

Heart Disease. By Sir William Broadbent. (London: Baillière, Tindall, and Cox. Fourth edition, 12s. 6d.)

The fourth edition is slightly enlarged, and contains more illustrations than the one which preceded it. Reference is made to experimental atheroma under the heading which deals with the pathology of aneurysm. The effect of the injection of adrenalin in some animals is very interesting, and, like every new fact, needs to be taken into consideration when the causation of a disease is being discussed upon which that fact may throw light. The discovery of a new cause for arterial disease ought to remind us, however, that there are other common causes of such disease of which comparatively little notice is taken. Extensive arterial disease may, for example, be occasionally seen in children under the age of one year who have died of diphtheria. This most commonly occurs, it is true, as an affection mainly of the inner coat, but the whole thickness of an artery may be diseased. We do not believe, however, that either disease of the arteries of this nature, or disease produced experimentally by injection of adrenalin, throw much light upon the causation of aneurysm. In another connection it is interesting to note that in a case of aneurysm which had perforated the pulmonary artery, the long, continuous murmur noted by other observers was present. The presence of such a murmur in these cases supports the opinion of Dr. G. A. Gibson that a similar murmur is diagnostic of patent ductus arteriosus.

Lectures on Auto-intoxication in Disease. By Ch. Bouchard. Translated by Thomas Oliver, M.D., F.R.C.P. (Philadelphia: F. A. Davis and Co.)

This second edition of Dr. Oliver's translation of the well-known work of Bouchard on auto-intoxication in disease has some concluding chapters by Dr. Oliver on the natural defences of the body against micro-organisms and toxins, and on auto-intoxication of intestinal origin. Dr. Oliver believes in a return to a simpler diet, to a diminution in the quantity of nitrogenous food taken, and a free use of milk. He also believes in the efficacy of intestinal antiseptics, and seems to favour salol, thymol, and naphthalin, Charrin is quoted as having stated that he has diminished the toxicity of the urine of patients suffering from chronic enteritis by two-thirds by giving 4 grams of beta-naphthol in 24 hours, and Surmount similarly diminished urinary toxicity by one-half. Intestinal antiseptics certainly seem to occasionally have the power to diminish flatulence and the pain or discomfort which results from it, but, judging from the clinical standpoint, their action is very uncertain. If, however, the toxicity of the urine is really largely dependent upon the presence in it of products of intestinal decomposition, and so-called intestinal antiseptics diminish the toxicity, their value can be said to be experimentally proved.

